# Effects of a multi-herbal extract on type 2 diabetes

**DOI:** 10.1186/1749-8546-6-10

**Published:** 2011-03-04

**Authors:** Jiyoung Yeo, Young-Mi Kang, Su-In Cho, Myeong-Ho Jung

**Affiliations:** 1 School of Korean Medicine, Pusan National University, Beomeo-ri, Mulguem-eup, Yangsan, Gyeongsangnam-do, 626-770, South Korea

## Abstract

**Background:**

An aqueous extract of multi-hypoglycemic herbs of *Panax ginseng *C.A.Meyer, *Pueraria lobata, Dioscorea batatas Decaisne, Rehmannia glutinosa, Amomum cadamomum Linné, Poncirus fructus *and *Evodia officinalis *was investigated for its anti-diabetic effects in cell and animal models.

**Methods:**

Activities of PPARγ agonist, anti-inflammation, AMPK activator and anti-ER stress were measured in cell models and in *db/db *mice (a genetic animal model for type 2 diabetes).

**Results:**

While the extract stimulated PPARγ-dependent luciferase activity and activated AMPK in C2C12 cells, it inhibited TNF-α-stimulated IKKβ/NFkB signaling and attenuated ER stress in HepG2 cells. The *db/db *mice treated with the extract showed reduced fasting blood glucose and HbA_1c _levels, improved postprandial glucose levels, enhanced insulin sensitivity and significantly decreased plasma free fatty acid, triglyceride and total cholesterol.

**Conclusion:**

The aqueous extract of these seven hypoglycemic herbs demonstrated many therapeutic effects for the treatment of type 2 diabetes in cell and animal models.

## Background

Caused by complex interactions of multiple factors, diabetes mellitus type 2 (type 2 diabetes) is characterized by decreased secretion of insulin by the pancreas and resistance to the action of insulin in various tissues (*eg *muscle, liver, adipose), leading to impaired glucose uptake [1]. Management of type 2 diabetes usually begins with change of diet and exercise [2] and most patients ultimately require pharmacotherapy, such as oral anti-diabetic drug (OAD) [1]. OADs include sulfonylurea, non-sulfonylurea secretagogues, biguanides (*eg *metformin), thiazolidinediones (*eg *TZD or glitazone) and glucosidase inhibitors and glucagon-like peptide-1 (GLP-1) inhibitor. All OADs, however, have adverse effects, *eg *weight gain with sulfonylurea, non-sulfonylurea secretagogues or TZD, edema and anemia with TZD [1].

A variety of medicinal herbal products including herbs used in Chinese medicine have beneficial effects on diabetes [3] and used as non-prescription treatment for diabetes [4]; many of these herbs have been formulated into multi-herbal preparation for enhanced effects [5]. While traditional formulae are often prescribed, their efficacy has yet to be investigated; recently, anti-diabetic multi-herbal formulae were studied and reported [6,7].

The present study reports a new anti-diabetic formula consisting of seven herbs, namely hypoglycaemic cadidates including *Panax ginseng *C.A.Meyer, *Pueraria lobata, Dioscorea batatas Decaisne, Rehmannia glutinosa *[8]*, Amomum cadamomum Linné *[9]*, Poncirus fructus *[10] and Evodia officinalis [11] which are available in South Korea. This formula's anti-diabetic molecular mechanisms and anti-hyperglycemic effects are demonstrated in cell models and *db/db *mice respectively.

## Methods

### Extract preparation

The dried herbs of *Panax ginseng *C.A. Meyer (Aralia family), *Pueraria lobata (Pea family), Dioscorea batatas DECAISNE (Dioscoreaceae), Rehmannia glutinosa (Phrymaceae), Amomum cadamomum Linné (Zingiberaceae), Poncirus fructus(Rhamnaceae)) *and *Evodia officinalis DODE(Rutaceae) *were purchased from Kwangmyungdang Natural Pharmaceutical (Korea) and identified morphologically, histologically and authenticated by Professor Su-In Cho (School of Korean Medicine, Pusan National University, Korea) according to standard protocol in National Standard of Traditional Medicinal Materials of The Korean Pharmacopeia [12]. Voucher specimens of all seven species were deposited in Pusan National University, Korea.

Powders of the herbs were mixed in equal amount (200 g each) and extracted in hot-water. The extract was freeze dried to powder and melt by dimethylsulfoxide (DMSO) when used. Macelignan, an active compound of *Myristica fragrans *Houtt (Myristicaceae), was prepared for positive control [13].

### Cell lines

Cell lines of human embryonic kidney (HEK) 293 (CRL-1573), 3T3-L1 pre-adipocytes (CL-173), HepG2 hepatocytes (HB-8065) and C2C12 skeletal myoblast cells (CRL-1772) were obtained from the American Type Culture Collection (ATCC, USA). HEK293 and HepG2 were cultured in Dulbecco's modified Eagle's medium (DMEM) containing glucose (Invitrogen, USA) supplemented with 10% (v/v) fetal bovine serum (Gibco BRL, USA). The 3T3-L1 pre-adipocytes were differentiated as described previously [14]. C_2_C_12 _skeletal myoblast cells were grown in DMEM supplemented with 2% horse serum to induce differentiation into myotubes.

### Reporter assays

The PPAR ligand-binding activity was measured with a GAL4/PPAR chimera assay and PPRE-tk-Luc reporter assay as described previously [15]. HEK293 cells were transfected with pFA-PPARγ and pFR-Luc (UAS-Gal4-luciferase) and treated with the extract, rosiglitazone (Alexis Biochemicals, USA) or macelignan at doses ranging from 2 to 10 μmol/L for 24 hours. For PPRE-tk-Luc reporter assay, HepG2 (2 × 10^5 ^cells/well) were transfected with PPRE-tk-Luc and incubated with the extract, rosiglitazone or macelignan for 24 hours. The luciferase activities were then determined with a luciferase assay system kit (Promega, USA).

To determine the anti-inflammatory activities and anti-endoplasmic recticulum (ER) stress, we transfected HepG2 cells (2 × 10^5 ^cells/well) with NFkB-Luc reporter or ERSE-Luc reporter using a Cignal™ Reporter Assay kit (SABiosciences, USA). The cells were then incubated with the extract, rosiglitazone or macelignan for 24 hours. The luciferase activities were determined with a Dual-Glo Luciferase assay system kit (Promega, USA).

### Real-time RT-PCR

We performed Real-time RT-PCR to determine the expression of adipose fatty acid-binding protein (aP2), acyl-CoA synthetase (ACS) and carnitine palmitoyltransferase-1 (CPT-1). The total RNA was extracted with TRIzol reagent (Invitrogen, USA) and subjected to reverse transcription with M-MLV Reverse Transcriptase (Promega, USA). The total RNA was then amplified (with gene-specific primers) and quantified with a fluorescence thermocycler (iQ™5, Multicolor Real-Time PCR System, Bio-Rad, USA).

### Western blot analysis

Total proteins were extracted with PRO-PREP reagent (iNtRON Biotechnology, Korea) and immuno-blotted with the antibodies of p-AMPK, IkBα, GRP78 or p-elf2α (Santa Cruz Biotechnology, USA) [15]. The immune complexes were identified with an enhanced chemiluminescence detection system (Amersham Biosciences, Sweden) according to the manufacturer's instructions and in conjunction with a Fluorochem gel image analyzer (MF-Chem:BIS 3.2, Alpha Innotech, USA).

### Animal study

Twenty-eight (28) male C57BL/KsJ-*db/db *mice aged 8 weeks were purchased from Jackson Laboratory (USA) and individually housed in polycarbonate cages under a 12-hour light-dark cycle at 21-23°C and 40-60% humidity. After a 2-week adaptation period, the body weight and fasting blood glucose level of the 10-week-old mice were measured. Then, the mice were equally divided into four groups (n = 7): (1) diabetic control, (2) rosiglitazone, (3) macelignan and (4) treatment (with the extract). All groups were fed a standard AIN-76 semi-synthetic diet (American Institute of Nutrition) and three experimental groups (rosigltiazone, macelignan and treatment) were orally administered with rosiglitazone (10 mg/kg body weight), macelignan (15 mg/kg body weight) or the extract (150 mg/kg body weight) for three weeks. After starved for 12 hours, the mice were anesthetized with ether and their blood samples were collected from the inferior vena cava for the measurement of the blood and plasma biomarkers such as HbA_1c _and insulin. All animal handlings during the experiments were in accordance with the Pusan National University guidelines for the care and use of laboratory animals.

### Fasting blood glucose, blood HbA_1c _and plasma biomarker analyses

During the experiments, the fasting blood glucose concentration was monitored by a Glucometer (GlucoDr, Allmedicus, Korea) with venous blood drawn from the mouse tail vein after a 12-hour fast. Moreover, the blood glycosylated hemoglobin (HbA_1c_) collected from sacrificed mice was measured with a MicroMat™ II Hemoglobin A_1c _Test (Bio-Rad Laboratories, USA). All blood samples obtained were centrifuged at 1000 × *g *for 15 min at 4°C for biochemical analysis. The plasma insulin, glucagon and C-peptide levels were measured with the enzyme-linked immunosorbent assay (ELISA) kits (ALPCO Diagnostics, USA).

Furthermore, the plasma lipids such as total cholesterol and triglyceride were determined with commercial kits (Sigma-Aldrich, USA) while the plasma free fatty acid (FFA) concentration was determined with an ACS (acyl-CoA synthetase)-ACOD(ascorbate oxidase) method (Wako Pure Chemical Industries, Japan).

### Intraperitoneal glucose tolerance test (IPGTT) and intraperitoneal insulin tolerance test (IPITT)

On the third week of treatment, an intraperitoneal glucose and insulin tolerance test (IPGTT and IPITT) were performed on all *db/db *mice after a 12-hour overnight fast. To determine the glucose and insulin tolerance, we injected the mice intraperitoneally with glucose (0.5 g/kg body weight) or insulin (2 unit/kg body weight). The glucose concentrations of blood drawn from the tail vein were determined immediately upon collection at 30, 60 and 120 min after glucose injection or at 30, 60 and 120 min after insulin injection.

### Statistical analysis

All statistical tests were two-sided, and the level of significance was set at 0.05. All data are presented as mean ± standard deviation (SD) for all groups. Statistical analyses were performed with the SPSS, version 18(SPSSInc., Chicago, IL, USA). One-way ANOVA(analysis of variance) with post-hoc test by Duncan's multiple-range test was used to examine differences among groups. The data were analyzed by Student's t-test for two group comparison.

## Results

### Effect on PPARγ agonist

**Figure 1:**
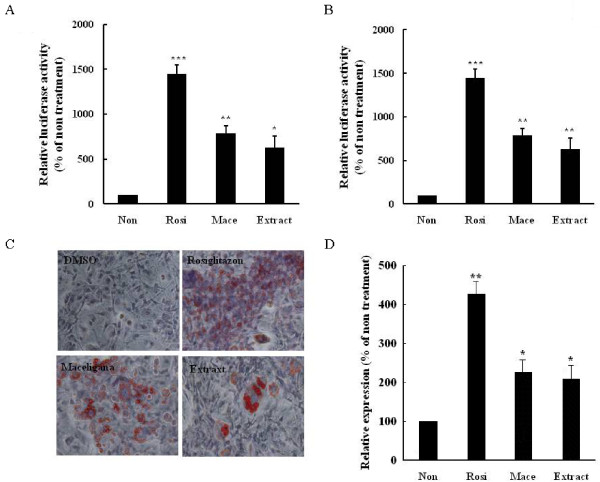
**Extract functions as a PPARγ agonist**. (A) Extract increased the ligand-binding activity of PPARγ. HEK293 cells were transfected with pFA-PPARγ and pFR-Luc (UAS-Gal4-luciferase) and then treated with extract (5 μg/ml), rosiglitazone (10 μM), or macelignan (10 μM) for 24 hours. (B) Extract induced transcriptional activity of PPARγ. Differentiated 3T3-L1 adipocytes were transfected with 3 × PPREs-tk-Luc and treated with extract (5 μg/ml), rosiglitazone (10 μM), or macelignan (10 μM) for 24 hours. (C) Extract induced adipogenesis. Oil red O staining was measured after differentiation of 3T3-L1 cells in medium containing 0.1% DMSO (control), extract (5 μg/ml), rosiglitazone (1 μM), or macelignan (10 μM) for seven days. (D) Extract increased PPARγ target gene (aP2) expression in 3T3-L1 adipocytes. Differentiated 3T3-L1 cells were treated with extract (5 μg/ml), rosiglitazone (10 μM), or macelignan (10 μM) for 24 hours. Expression of mRNAs was estimated using quantitative real-time RT-PCR, and the results were expressed as mRNA levels relative to 0.1% DMSO (control). Data represent are shown as mean ± SD of three independent experiments (*P < 0.05, **P < 0.01, ***P < 0.001)

To determine if the extract was a PPARγ agonist, we searched the cell-based GAL4/PPAR chimera transactivation in Hek293 cells. As shown in Figure 1A, the extract increased the PPARγ-dependent luciferase activity (*P *= 0.035 *vs *non-treatment) similar to that of rosiglitazone (*P *= 0.001 *vs *non-treatment), a well-known PPARγ agonist, and macelignan (*P *= 0.005 *vs *non-treatment), a PPARα/γ dual agonist used as positive control throughout the experiments. To further explore the PPARγ agonist potential of the extract, transient transfections were performed in differentiated 3T3-L1 adipocytes with the tk-luciferase vector containing PPAR-responsive elements (PPREs) and then treated with the extract. The treatment stimulated PPRE-dependent luciferase activities in transfected cells (*P *= 0.005 *vs *non-treatment) (Figure 1B). To provide biological evidence that the extract is a PPARγ ligand, we investigated adipocyte differentiation and expression of the adipocyte marker gene in 3T3-L1 cells treated with the extract. The treatment led to a significant increase in the formation of lipid droplets in similar to rosiglitazone and macelignan (Figure 1C). Moreover, the extract increased the expression of adipose fatty acid-binding protein (aP2) (*P *= 0.042 *vs *non-treatment) (Figure 1D). Taken together, these results demonstrated that the extract was a PPARγ agonist.

### Effect on AMPK activation

**Figure 2:**
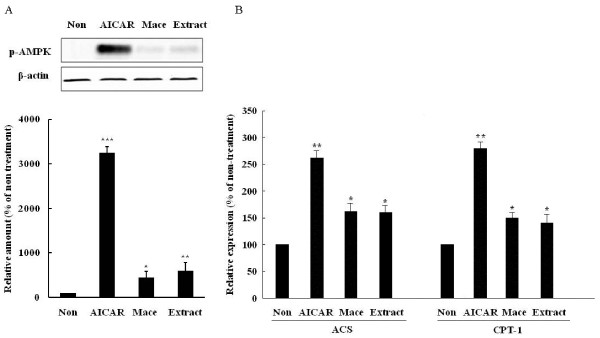
**Extract activates AMPK in C2C12 cells**. (A) Extract increased AMPK phosphorylation. C2C12 cells were treated with aminoimidazole-4-carboxamide-1-β-d-ribofuranoside (1 mmol/l), extract (5 μg/ml), or macelignan (10 μM) for 24 hours. Phosphorylated AMPK was examined by Western blot analysis, (B) extract increased the mRNA expression of ACS, CPT-1. The expression was estimated using quantitative real-time RT-PCR. Data represent are shown as mean ± SD of three independent experiments (*P < 0.05, **P < 0.01, ***P < 0.001)

To determine if the extract mediated the AMP-activated protein kinase (AMPK) activation, we measured the AMPK phosphorylation and expression of fatty acid oxidation genes in C_2_C_12 _cells incubated with the extract. As with the AMPK activator, aminoimidazole-4-carboxamide-1-β-d-ribofuranoside (AICAR) (*P *= 0.001 *vs *non-treatment), the treatment activated AMPK in C_2_C_12 _cells (*P *= 0.007 *vs *non-treatment), similar to when samples were treated with macelignan (*P *= 0.042 *vs *non-treatment) (Figure 2A). Consistent with the results of AMPK phosphorylation, the treatment increased the expression of acyl-CoA synthetase (ACS) (*P *= 0.048 *vs *non-treatment) and carnitine palmitoyltransferase-1(CPT-1) (*P *= 0.041 *vs *non-treatment) (Figure 2B), suggest that the extract activated AMPK.

### Effect on inflammatory processes

**Figure 3:**
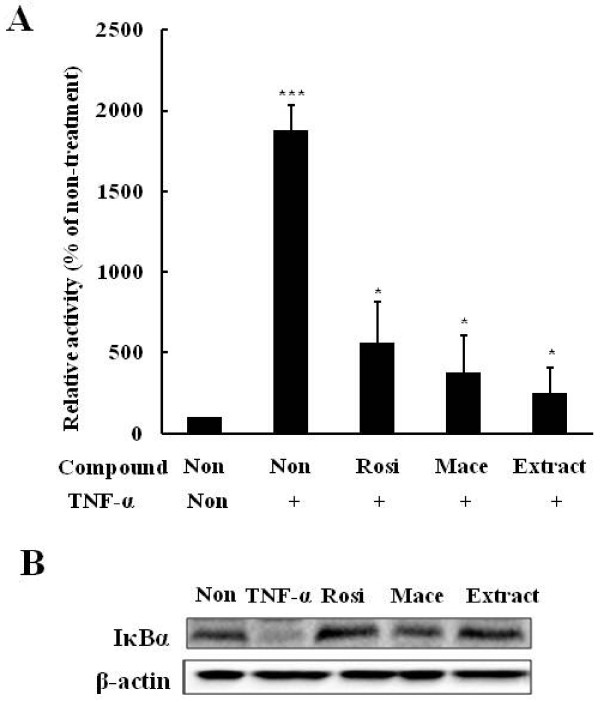
**Extract inhibits NFkB signaling in HepG2 cells**. (A) extract prevented the increase of TNF-α-stimulated luciferase activity in TNF-α treated HepG2. HepG2 cells were transfected with NFkB-Luc reporter and then treated with extract (5 μg/ml), rosiglitazone (10 μM), or macelignan (10 μM) for 24 hours in the presence of TNF-α (10 ng/ml) (B) extract increased the IkB level. HepG2 cells were preincubated with extract (5 μg/ml), rosiglitazone (10 μM), or macelignan (10 μM) for 24 hours and then treated with TNF-α (10 ng/ml) for one hour. IκBα was measured by Western blot analysis. Data represent are shown as mean ± SD of three independent experiments (*P < 0.05, **P < 0.01, ***P < 0.001)

As inflammatory processes play potential roles in the pathogenesis of insulin resistance, we investigated whether the extract possessed anti-inflammatory effects, including the inhibitory effects of the extract on IKKβ/NFkB signaling in HepG2 cells treated with TNF-α using NFkB response element containing reporter. While TNF-α treatment increased the NFkB-dependent luciferase activity (*P *= 0.001 *vs *non-treatment), The extract effectively prevented this increase (*P *= 0.034 *vs *TNF-α treatment) (Figure 3A). Furthermore, the extract increased the IkBα level reduced by TNF-α treatment, which was consistent with rosiglitazone and macelignan (Figure 3B). These results indicated that the extract exerted anti-inflammatory effects.

### Effect on attenuation of ER stress

**Figure 4:**
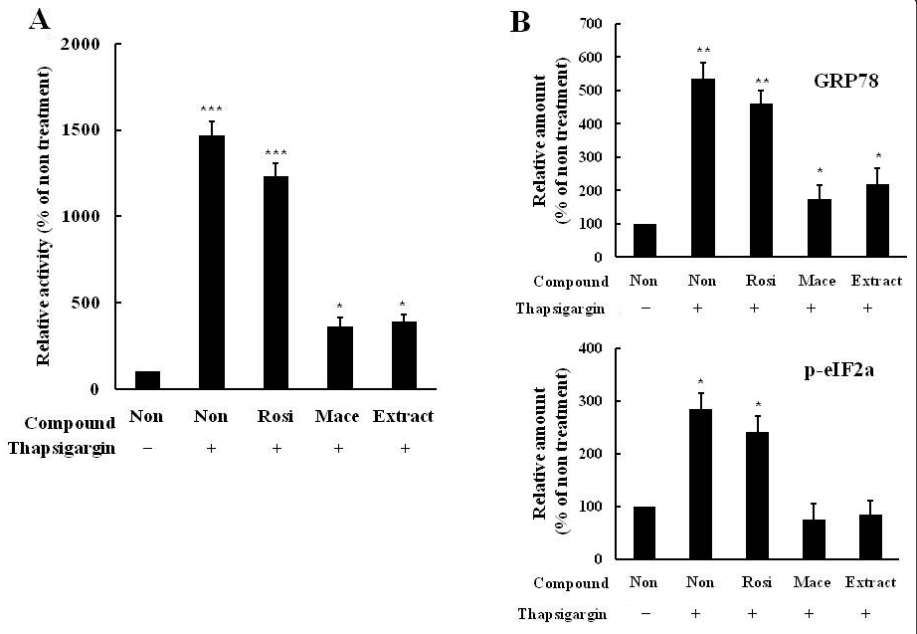
**Extract attenuates the induction of ER stress**. (A) Extract prevented the increase of thapsigargin-stimulated luciferase activity in thapsigargin-treated HepG2. HepG2 cells were transfected with ERSE-Luc reporter and then treated with extract (5 μg/ml), rosiglitazone (10 μM), or macelignan (10 μM) for 24 hours in the presence of thapsigargin (10 ng/ml). (B) Extract increased the levels of GRP78 and peIF. HepG2 cells were preincubated with extract (5 μg/ml), rosiglitazone (10 μM), or macelignan (10 μM) for 24 hours and then treated with thapsigargin (10 ng/ml) for 24 hours. GRP78 p-eIF were measured by Western blot analysis. Data represent are shown as mean ± SD of three independent experiments (*P < 0.05, **P < 0.01, ***P < 0.001)

It has been recently suggested that ER stress plays a central role in the development of insulin resistance and diabetes by impairing insulin signaling through c-Jun NH_2_-terminal kinase (JNK) activation [16]. Therefore, we investigated whether the extract inhibited ER stress. We first examined the inhibitory effects on the luciferase activity of ERSE response element containing reporter in HepG2 cells treated with the ER stress inducer, thapsigargin. While thapsigargin treatment increased the ERSE-dependent luciferase activity (*P *= 0.001 *vs *non-treatment), the extract effectively blocked the thapsigargin-mediated stimulation (*P *= 0.039 *vs *thapsigargin treatment) (Figure 4A). When ER stress indicators such as GRP78 and p-elF2α were examined in thapsigargin-treated HepG2 cells, Treatment by the extract suppressed the increase of the indicators by thapsigargin (*P *= 0.045 *vs *thapsigargin treatment) (Figure 4B). Taken together, these results demonstrated that the extract exerted protective effects against ER stress.

### Effects on body weight change and fasting blood glucose in db/db mice

**Figure 5:**
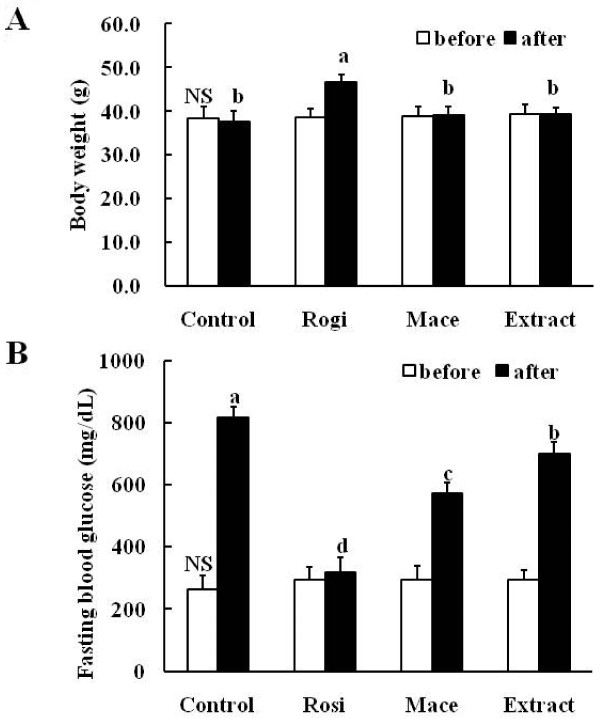
**Extract beneficial effects on (A) body weight and (B) fasting blood glucose level in *db/db *mice after 3-week treatment**. Values shown are mean ± SD (*n *= 7). ^abcd ^Data not sharing a common letter are significantly different (*P *< 0.05) after one-way ANOVA and Duncan's multiple-range test. NS: non-significance

To examine the *in vivo *anti-diabetic effects of the extract on diabetes, we orally administered rosiglitazone (10 mg/kg), macelignan (15 mg/kg) and the extract (150 mg/kg) to C57BL/KsJ-*db/db *mice every day for three weeks and the extract's effects were compared with rosiglitazone and macelignan. Treatment with the extract did not have a significant effect on the body weights in the *db/db *mice; however, mice treated with rosiglitazone had final body weights significantly higher than those of the others (*P *= 0.001 *vs *control) (Figure 5A). The baseline (day 0) fasting blood glucose levels did not differ between groups; however, at the end of the experiment, the values of the extract-treated group were significantly lower compared to the diabetic control group (*P *= 0.022 *vs *control) and so did the other groups treated with rosiglitazone (*P *= 0.001 *vs *control) and macelignan (*P *= 0.002 *vs *control). The blood glucose levels of the extract-treated mice were significantly reduced by about 15% compared to the control (Figure 5B).

### Effects on postprandial glucose and insulin sensitivity in db/db mice

**Figure 6:**
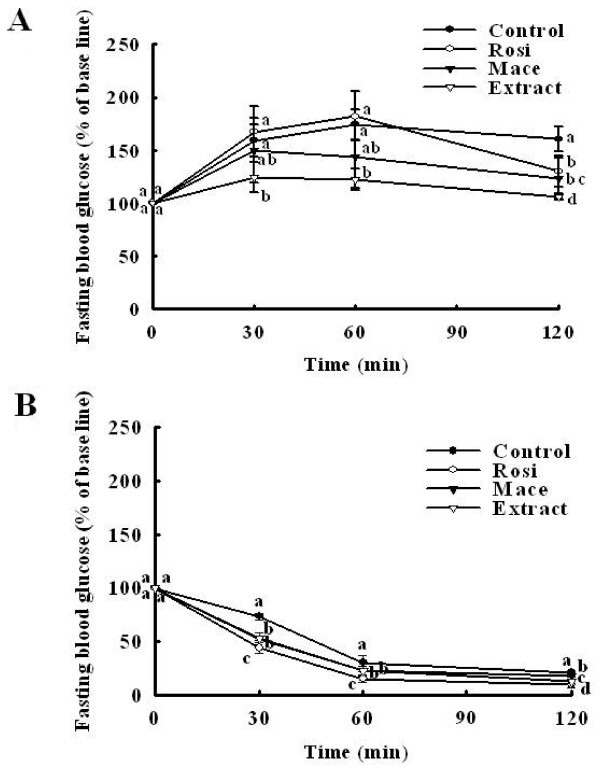
**Extract improved (A) postprandial glucose and (B) insulin sensitivity in *db/db *mice**. After a 12-hourfast, male mice (12 weeks-old) were intraperitoneally injected with glucose (0.5 g/kg body weight) and insulin (2 units/kg body weight). The blood glucose concentration was then measured at the indicated times and was presented as a percentage of the glucose injection zero time. Values are mean ± SD (*n *= 7). ^abcd ^Data not sharing a common letter are significantly different (*P *< 0.05) after one-way ANOVA and Duncan's multiple-range test.

To assess glucose homeostasis and insulin sensitivity in db/db mice treated with the extract, we performed glucose tolerance and insulin tolerance tests before the end of the experiment. As shown in Figure 6A, the extract significantly reduced the blood glucose levels (*P *= 0.001 *vs *control) similar to rosiglitazone (*P *= 0.003 *vs *control) and macelignan (*P *= 0.004 *vs *control) used as positive controls compared with the diabetic control groups. The insulin tolerance test also showed that reduction in blood glucose levels in response to insulin was much greater in mice treated with the extract than in untreated *db/db *mice (*P *= 0.002 *vs *control) (Figure 6B). These findings indicate that treatment with the extract affected not only regulation of the postprandial glucose level, but also enhanced the insulin sensitivity.

### Effects on plasma lipids in db/db mice

Effects of the extract on plasma triglycerides and FFAs levels and total cholesterol were investigated. Specifically, treatment with the extract significantly decreased the plasma free fatty acid (*P *= 0.021 *vs *control), triglyceride (*P *= 0.012 *vs *control) and total cholesterol (*P *= 0.003 *vs *control) concentrations of the diabetic control *db/db *mice compared with untreated *db/db *mice when the experiment ended (Table 1). As lipolysis and circulating free fatty acids increase under insulin resistance conditions, these results demonstrate that the decrease in plasma lipids may contribute to the improvement of severe diabetes, at least partially.

**Table 1:** Effects of the extract on the plasma lipid profiles in *db/d b *mice

	Control	Rosiglitazone	Macelignan	Extract
FFAs (mmol/L)	2.28 ± 0.21^a^	0.94 ± 0.05^c^	1.70 ± 0.21^b^	1.75 ± 0.11^b^
Triglyceride (mg/dL)	296.2 ± 59.5^a^	109.4 ± 29.2^c^	259.0 ± 54.9^ab^	217.9 ± 34.9^b^
Total cholesterol (mg/dL)	146.1 ± 15.0^b^	181.9 ± 5.84^a^	110.0 ± 22.4^c^	119.4 ± 3.41^c^

^abc ^Data in the same row not sharing a common superscript indicate a significant difference (*P *< 0.05) between groups after one-way ANOVA and Duncan's multiple-range test; mean ± SD (*n *= 7); FFAs: free fatty acids.

### Effects on glycosylated hemoglobin level and plasma biomarkers in db/db mice

Mice receiving the treatment with the extract showed a significantly lower blood glycosylated hemoglobin level compared to the diabetic control *db/db *mice (*P *= 0.002 *vs *control). Both the plasma insulin (*P *= 0.042 *vs *control) and C-peptide levels (*P *= 0.038 *vs *control) were significantly higher in the extract-treated *db/db *mice than in the diabetic control *db/db *mice; however, the glucagon levels were significantly lower than those of the diabetic control *db/db *mice (*P *= 0.018 *vs *control). Therefore, treatment with the extract significantly improved the ratio of insulin/glucagon (I/G) when compared to the diabetic control *db/db *mice (Table 2).

**Table 2:** Effects of the extract on concentrations of blood and plasma biomarkers in *db/db *mice

	**HbA**_**1c **_**(%)**	Insulin (ng/mL)	Glucagon (ng/mL)	C-peptide (ng/mL)	I/G
**Control**	10.7 ± 0.46^a^	1.48 ± 0.89^b^	0.37 ± 0.07^a^	3.12 ± 0.73^b^	4.68 ± 1.11^b^
**Rosiglitaozne**	7.40 ± 0.88^c^	3.43 ± 1.05^a^	0.32 ± 0.02^a^	4.76 ± 1.09^a^	9.67 ± 3.05^ab^
**Macelignan**	10.8 ± 0.25^a^	1.52 ± 0.12^b^	0.23 ± 0.05^b^	4.14 ± 0.35^ab^	6.74 ± 1.31^b^
**Extract**	9.3 ± 0.80^b^	3.15 ± 1.43^a^	0.21 ± 0.02^b^	4.79 ± 0.44^a^	14.2 ± 7.55^a^

^abc ^Data in the same row not sharing a common superscript indicate a significant difference (P < 0.05) between groups after one-way ANOVA and Duncan's multiple-range test; mean ± SD (*n *= 7);HbA_1c_: blood glycosylated hemoglobin; I/G: ratio of insulin/glucagon.

## Discussion

In this study, we tested a formulation of seven medicinal herbs including *Panax ginseng *C.A.Meyer for the anti-diabetic effects in cells and in vivo. We found that the extract from the seven herbs functioned as PPARγ agonists and an AMPK activators, as well as inhibitors of inflammation and ER stress. PPARγ can improve insulin sensitivity and glucose tolerance by regulating lipid storage, glucose homeostasis and adipokine regulation [17]. The TZD group, especially rosiglitazone and troglitazone, are agonists of PPARγ [18]. The extract significantly increased the PPARγ-dependent luciferase activity *in vitro *and stimulated the formation of lipid droplets and the expression of aP2 upon transient transfection of 3T3-L1 cells. Rb1, the most abundant ginsenoside in ginseng root, increases the expression of mRNA and protein of PPARγ and exerts anti-diabetic and insulin-sensitizing activities [19]. 20(S)-protopanaxatriol (PPT), a ginsenoside metabolite, increases PPARγ-transactivation activity with an activity similar to troglitazone, and up-regulates the expression of PPARγ target genes such as aP2, LPL and PEPCK [15]. Therefore, the activity of PPARγ against may be due to *Panax ginseng*. Further studies are required to confirm this speculation.

Activation of AMPK enhances insulin sensitivity through increased glucose uptake and lipid oxidation in skeletal muscle and inhibition of glucose and lipid synthesis in the liver [20]. Metformin acts as an activator of AMPK in the liver and skeletal muscle [21]. The present study demonstrated that the extract activated AMPK in C2C12 and induced increased expression of AMPK target genes. Ginsenoside Rh2 and Rg3, a red ginseng rich constituent, activates AMPK significantly in 3T3-L1 adipocytes and to contribute to antiobesity effects [22,23]. Further studies are required to characterize which herb activates AMPK.

Inflammatory cytokines and IKK attenuate insulin signaling through serine phosphorylation of IRS-1 [24]. High doses of salicylates, which block the IKKb activity, ameliorate hyperglycemia and insulin resistance in diabetes and obesity [25]. Our results showed that the extract effectively suppressed NFkB-dependent luciferase activity in TNF-α-treated HepG2 cells and increased the IkB level, suggesting that the extract blocked the activation of the NF-κB pathways.

By activating c-Jun amino-terminal kinase (JNK), which induces insulin resistance in liver and skeletal muscle and inhibits beta cell function, ER stress induces the development of type 2 diabetes [26]. Thus, agents that alleviate ER stress may act as potent anti-diabetic agents. Chemical or biological compounds such as macelignan [27], chromium-phenylalanine [28], PBA (phenyl butyric acid) [29] or TUDCA (tauroursodeoxycholic acid) [30] or molecular chaperon have been shown to inhibit ER stress and enhance insulin sensitivity, thereby normalizing hyperglycemia. The present study found that the extract alleviated ER stress and efficiently suppressed ERSE-dependent transactivation in thapsigargin-treated HepG2 and expression of ER stress marker proteins. In future studies, we will determine the optimal combination ratio for this formulation and isolate its active fractions.

## Conclusion

The aqueous extract of these seven hypoglycemic herbs demonstrated anti-diabetic effects on type 2 diabetes.

## Abbreviations

ACS: acyl-CoA synthetase; AICAR: aminoimidazole carboxamide ribonucleotide; AMPK: AMP-activated protein kinase; aP2: adipose fatty acid-binding protein 2; CPT-1: carnitine palmitoyltransferase-1; DMSO: Dimethylsulfoxide; ER: endoplasmic reticulum; ERSE: ER stress response element; FFAs: free fatty acids; eIF: elongation initiation factor; GLP-1: glucagon-like peptide-1; HbA_1c_: blood glycosylated hemoglobin; HDL-cholesterol: high density lipoprotein-cholesterol; HEK293: human embryonic kidney293; IKK: IκB kinase; IPGTT: intraperitoneal glucose tolerance test; IPITT: intraperitoneal insulin tolerance test; JNK: c-Jun N-terminal kinases; LPL: lipoprotein lipase; OAD: oral antidiabetic drug; PBA: phenyl butyric acid; PPAR: peroxisome proliferator-activated receptor; PPREs: PPAR-responsive elements; SD: standard deviation; TZD: thiazolidinedione

## Competing interests

The authors declare that they have no competing interests.

## Authors' contributions

MHJ designed the study and wrote the manuscript. SIC prepared the aqueous extract from the herbs. JY conducted the *in vivo *experiments. YMK conducted the experiments in cultured cells. All authors read and approved the final version of the manuscript.
